# Quantitative analysis of differentially expressed proteins in psoriasis vulgaris using tandem mass tags and parallel reaction monitoring

**DOI:** 10.1186/s12014-020-09293-8

**Published:** 2020-08-12

**Authors:** Yu Li, Peng Lin, Siyao Wang, Shuang Li, Rui Wang, Lin Yang, Hongmei Wang

**Affiliations:** 1 Department of Dermatology, Tianjin Academy of Traditional Chinese Medicine Affiliated Hospital, Tianjin, People’s Republic of China; 2 Tianjin University of Traditional Chinese Medicine, Tianjin, People’s Republic of China; 3 Shenzhen Luohu District Hospital of Traditional Chinese Medicine, Shenzhen, Guangdong People’s Republic of China

**Keywords:** Differentially expressed proteins, Psoriasis vulgaris, Drug metabolism pathway, Quantitative proteomics

## Abstract

**Background:**

*Psoriasis vulgaris* (PV) is a chronic autoimmune inflammatory disease with epidermal hyperkeratosis and parakeratosis.

**Methods:**

The study was to elucidate the pathogenesis of PV by quantitative proteomic analysis of skin lesion biopsies of PV and healthy tissues with tandem mass tags (TMTs) coupled with liquid chromatography–mass spectrometry (LC–MS)/MS.

**Results:**

A total of 4562 differentially expressed proteins (DEPs) between PV lesional tissues (n = 11) and healthy tissues (n = 11) were identified, of which 299 were upregulated and 206 were downregulated using |fold change| > 1.3 as the cutoff threshold. The Gene Ontology (GO) annotation and Kyoto Encyclopedia of Genes and Genomes (KEGG) pathway enrichment analysis revealed that the DEPs were mainly enriched in the activation of immune cells (drug metabolism pathway, NOD-like pathway, and IL-17 pathway), cell proliferation (ribosomal pathway, DNA replication pathway, and base replication pathway), metabolism-related pathways (fatty acid biosynthesis and metabolism, PPAR pathway, glycerophospholipid metabolism, and cortisol synthesis and breakdown), and glandular secretion (saliva secretion, gastric acid secretion, and pancreatic fluid secretion). Thirteen DEPs that were relatively highly expressed in the drug metabolism pathway were validated with parallel reaction monitoring (PRM), of which MPO, TYMP, IMPDH2, GSTM4, and ALDH3A1 were highly expressed in PV, whereas CES1, MAOB, MGST1, and GSTT1 were less expressed in PV.

**Conclusions:**

These findings confirmed that these proteins participate in the drug metabolism-other enzyme pathways and play crucial roles in the activation and proliferation of immune cells in the pathogenesis of PV.

## Background

Psoriasis vulgaris (PV) is an immune-related chronic inflammatory skin disease [1] characterized by well-demarcated, erythematous, and scaly papule. Several exogenous and endogenous factors, including genetics and environmental factors together with infection, immunity, and endocrine levels, have been observed to trigger the occurrence and development of PV. In recent years, the incidence rates of PV have consistently increased year by year [2, 3]. PV has long been considered a systemic inflammatory disease [4] mediated by the cells and molecules of both the innate and adaptive immune systems [5], with the participation of keratinocytes (KCs), dendritic cells (DCs), T lymphocytes, and neutrophils causing the proliferation and differentiation of keratinocytes as well as dermal vascular proliferation and expansion. Epidemiology analyses have previously demonstrated a significant association between PV and metabolic syndrome, which includes obesity, hyperlipidemia, and diabetes [6]. Although many treatments are available to reduce the symptoms and appearance of PV, its pathogenesis remains unknown and no definite cure exists.

In recent years, the development of multiple “-omic”-based approaches has provided an effective way to identify and characterize proteins that are involved in the pathogenesis of PV. Swindell et al. [7] used the method of Label-free GeLC–MS/MS and LTQ-Orbitrapnano LC–MS/MS finding that 748 proteins had differential levels between psoriatic lesional and non-lesional biopsies, including those with concordant and discordant mRNA changes, and most of which were targeted by IL-17A.Tandem mass tags (TMTs) are used in a quantitative proteomic approach that allows the quick identification and quantitation of thousands of proteins [8–10] to better understand the pathogenesis of diseases. However, very few studies have examined the skin of PV lesions at the proteome level. Recently, we characterized differentially expressed proteins (DEPs) with TMT-based quantitative proteomic analysis of peripheral blood mononuclear cells from patients with PV [11]. Kyoto Encyclopedia of Genes and Genomes (KEGG) pathway enrichment analysis revealed the involvement of DEPs in the oxidative phosphorylation pathway, the tricarboxylic acid cycle, and the nuclear factor (NF)-κB pathway in our previous study. Besides, we also annotated some significant proteins which was closely associated with psoriasis, for example, MPO, TYMP, IMPDH2, ALDH3A2, GSTM4, S100A8/9, COX5A, CD40, HLA-A, FABP5, STAT1/2/3, IL-18, etc. Proteins with a differential expression between PV lesions and normal tissue can be considered as pathological biomarkers for therapeutic development. Therefore, TMT-based quantitative proteomic analysis provides new avenues for better understanding of the pathogenesis of PV at the protein level.

In the present study, TMT-based quantitative proteomic analysis was performed to identify DEPs by comparing the skin lesions of PV patients and heathy tissues. PRM was first used to quantitatively verify DEPs in skin lesions of PV patients and healthy human tissues. The findings will provide a fundamental basis for exploring the pathogenesis of PV and specific proteins as potential therapeutic targets.

## Materials and methods

### Sample collection and ethics statement

Skin lesional tissues were sampled from 11 PV patients (4 males and 7 females; average age, 34 ± 10 years; mean severity index, 9.2 ± 1.8 points) from November 2017 to April 2018 in the Outpatient Department of the Tianjin Academy of Chinese Medicine. Patients were diagnosed as having PV according to the diagnostic criteria described by *Textbook of Dermatology* [12] (Additional file 1: Table S1). The details of the patients of their pathological sections were shown in Additional file 2: Fig S1. In addition, normal tissues were sampled from 11 healthy individuals without PV (2 males and 9 females; average age, 34 ± 10 years) in the Outpatient Department as the control group. Statistical analysis with IBM SPSS Statistical Version 24 (Armonk, NY) showed no significant differences in age or gender between the case and control groups. The tissue specimens were washed with phosphate-buffered saline (2–4 °C, pH 7.2–7.4), placed in 5-mL Eppendorf tubes, immediately frozen in liquid nitrogen for 5–10 min, and stored at -80 °C. All subjects provided a written informed consent prior to sample collection.

### Protein extraction and trypsin digestion

Tissue specimens were quickly frozen in liquid nitrogen, pulverized into a fine powder, and added to four volumes of lysis buffer (8 M urea, 1% protease inhibitor, 2 mM EDTA protein lysate) for ultrasound lysis at 4 °C, followed by centrifugation at 12,000×*g* for 10 min. The supernatant was transferred to determine the protein concentration with a bicinchoninic acid assay kit (Bìyúntiān, Shanghai, China). The proteins were reduced with 5 mM dithiothreitol protein solution at 56 °C for 30 min, and then iodoacetamide was added to a final concentration of 11 mM for incubation in the dark at room temperature for 15 min. The samples were diluted to a urea concentration < 2 M, and trypsin (trypsin:protein = 1:50) was added for overnight digestion at 37 °C, followed by an additional trypsin (trypsin:protein = 1:100) digestion for 4 h.

### TMT labeling

The lysed peptides were desalted with StrataXC18 (Phenomenex, Torrance, CA, USA) and lyophilized *in vacuo*. The peptides were dissolved in 0.5 M tetraethylammonium bromide. The peptides from the psoriasis group (pso) and the normal group (con) were divided into three groups, respectively, and labeled according to the instructions of the TMT kit (Thermo, Chicago, IL, USA): 126 is con_1, 127 is con_2, 128 is con_3, 129 is pso_1, 130 is pso_2, and 131 is pso_3.

### High-performance liquid chromatography (HPLC) fractionation

In total, 60 fractions were fractionated from the peptides of each sample in an Agilent 300 Extend C18 column (5 μm, 4.6 × 250 mm) using a gradient of 8% to 32% acetonitrile (pH 9) for 60 min. The eluents of 18 fractions were combined for lyophilization *in vacuo*.

### Mass spectrometry quality control detection

Mass error of all identified peptides were examined (Additional file 3: Fig S2A), which were centered at 0 and are concentrated in the range below 10 PPM. Most peptide length were between 8 and 20 amino acid residues (Additional file 3: Fig S2B), which conformed to the law of trypsin digestion of peptide segment.

### Liquid chromatography–mass spectrometry (LC–MS)/MS analysis

The peptides were dissolved in mobile phase A for liquid chromatography and separated using the EASY-nLC1000 ultrahigh-performance liquid system. Mobile phase A consisted of aqueous 0.1% formic acid and 2% acetonitrile, whereas mobile phase B consisted of 0.1% formic acid and 90% acetonitrile. The liquid chromatography gradient was as follows: 0–20 min, 9–23% B; 20–33 min, 23–35% B; 33–37 min, 35–80% B; 37–40 min, 80% B; the flow rate was 700 nL/min. The separated peptides were injected into the nanospray ionization (NSI) ion source for ionization at a voltage of 2.0 kV and then analyzed by Q ExactiveTM Plus mass spectrometry. Peptide precursor ions and their secondary fragments were detected and analyzed using a high-resolution Orbitrap instrument. The first-order mass spectra (MS1) were captured using a scan range of 350–1800 *m/z* with a scan resolution of 70,000, and the second-order mass spectra (MS2) were received with a fixed scan range of 100 *m/z* and a secondary scan resolution of 17,500. For data acquisition mode, a data-dependent scanning program was used for secondary mass spectrometric analysis. The automatic gain control (AGC) was set to 5E4, the signal threshold was 10,000 ions/s, and the maximum injection time was 200 ms. Precursor ions were excluded from rescanning with 30 s of dynamic exclusion time of tandem mass scanning.

### Protein identification and database search

The MS data were searched in the protein sequence database SwissProt Human (20,317 sequences) using Maxquant (v1.5.2.8). The parameter settings were as follows: digestion method, trypsin/P; number of missed cut sites, 2; minimum peptide length, 7 amino acid residues; first-level precursor ion mass tolerance of the first search and the main search, 20 ppm and 5 ppm, respectively; and secondary fragment mass tolerance, 0.02 Da. The quantitative method was set to TMT-6plex with a false discovery rate of 1% for the identification of protein and peptide-to-spectrum matches.

### PRM

The peptides were separated by an ultrahigh-performance liquid system and injected into an NSI ion source for ionization for analysis with Q ExactiveTM mass spectrometry with the following settings: primary mass spectrometer AGC, 3E6; maximum ion implantation time (IT), 50 ms; secondary mass spectrometer AGC, 1E5; maximum IT, 120 ms; and isolation window, 1.6 *m/z*. The peptide parameters were as follows: protease, trypsin [KR/P]; maximum number of missed cleavage sites, 0; peptide length, 7–25 amino acid residues; and cysteine alkylation, fixed modification. The transition parameters were as follows: precursor ion charge, 2, 3; product ion charge, 1; and ion type, b, y. Fragment ion selection started from the third to the last, while debris ion selection started from the third to the last, with an ion-matched mass tolerance of 0.02 Da. The original data of PRM was in Additional file 4: Fig S3.

## Results

### Identification of DEPs

In total, 4562 proteins were identified, of which 3648 proteins had quantitative information and annotation terms (Additional file 5: Table S2). In addition, 505 DEPs were characterized using |fold-change| > 1.3 as the cutoff criterion, of which 299 DEPs were upregulated and 206 DEPs were downregulated.

### Subcellular structure classification of DEPs

**Fig. 1:**
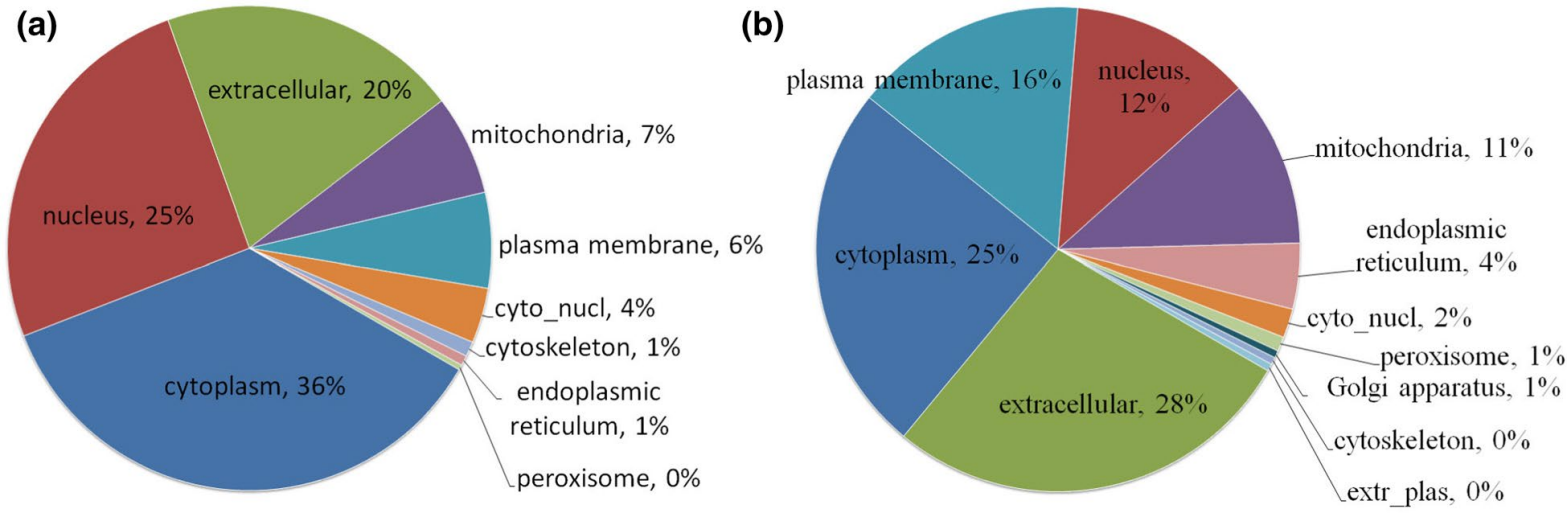
Subcellular localization of DEPs. **a** Upregulated DEPs. **b** Downregulated DEPs

As shown in Fig. 1, the upregulated DEPs were mainly localized in the cytoplasm (36%), nucleus (25%), extracellular space (20%), mitochondria (7%), and cell membrane (6%) (Fig. 1a); whereas the downregulated DEPs were mainly localized in the extracellular space (28%), cytoplasm (25%), cell membrane (16%), nucleus (12%), and mitochondria (11%).

### GO analysis of DEPs

**Fig. 2:**
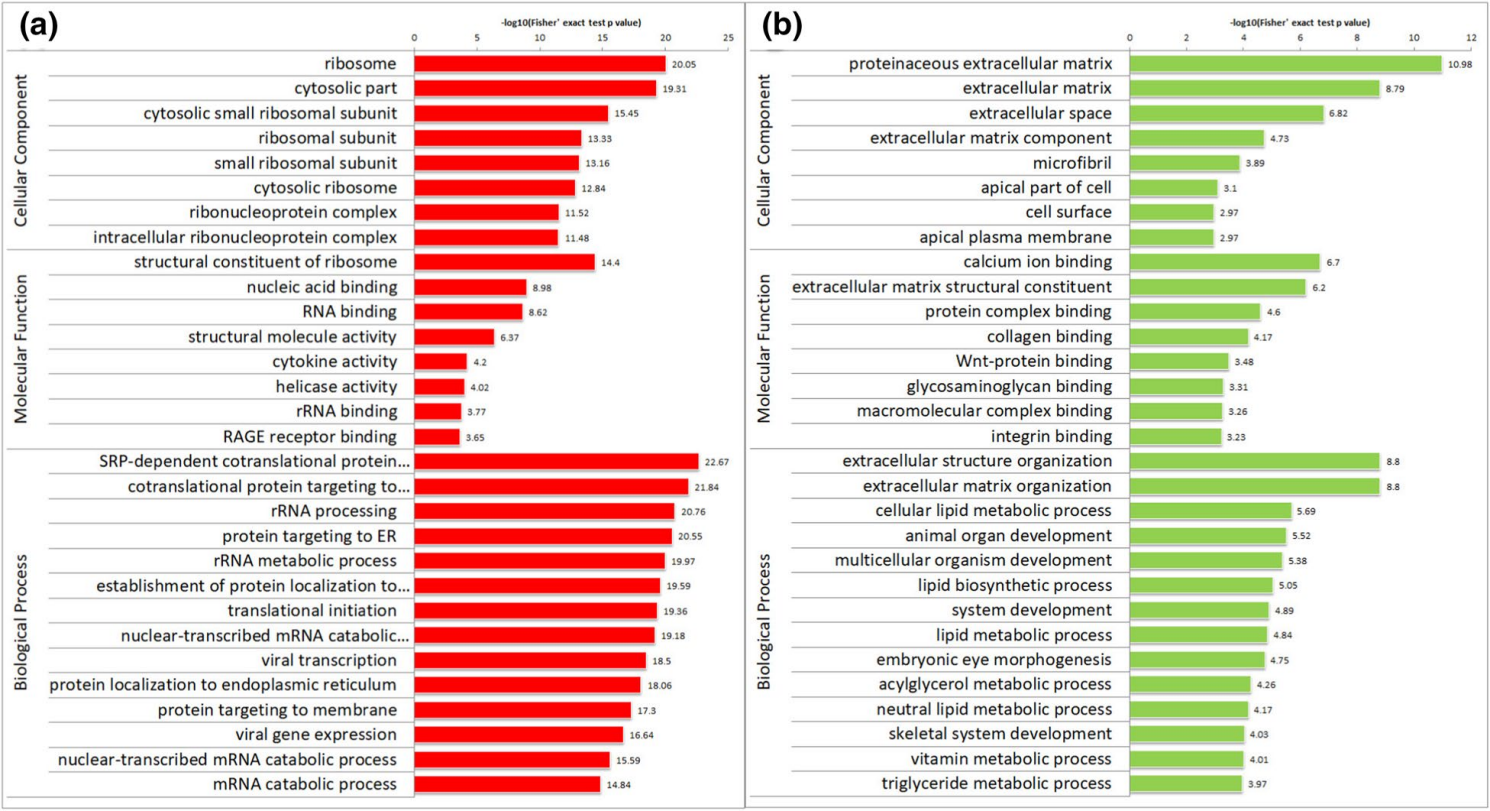
GO annotation of 505 DEPs (pso/con). **a** Upregulated DEPs. **b** Downregulated DEPs. The values on the horizontal axis are negative logarithmic conversions of significant P values (P < 0.05)

The DEPs were assigned by GOSeq (vision Release2.12) into three groups: biological process (BP), cellular component (CC), and molecular function (MF). As shown in Fig. 2, the upregulated DEPs were mainly enriched in the ribosomes, cytoplasm, cytoplasmic small ribosomal subunits, and ribosomal subunits; whereas the downregulated DEPs were mainly assigned to the extracellular matrix, cytoskeleton, and cell membrane in the CC category. In the MF category, the upregulated DEPs were enriched in the functions of ribosome structure composition, nucleic acid binding, RNA binding, and structural molecular activity; whereas the downregulated DEPs were mainly assigned to Ca^2+^ binding, extracellular matrix structure composition, protein complex binding, and collagen binding. In the BP category, the upregulated DEPs were mainly assigned to ribosome activation, rRNA processes, endoplasmic reticulum protein synthesis, nuclear transcriptional mRNA catabolism, and other processes; whereas the downregulated DEPs were mainly enriched in extracellular tissues, cellular lipid metabolism, animal organ development, multicellular organisms, and lipid biosynthesis.

### KEGG pathway enrichment analysis of DEPs

**Fig. 3:**
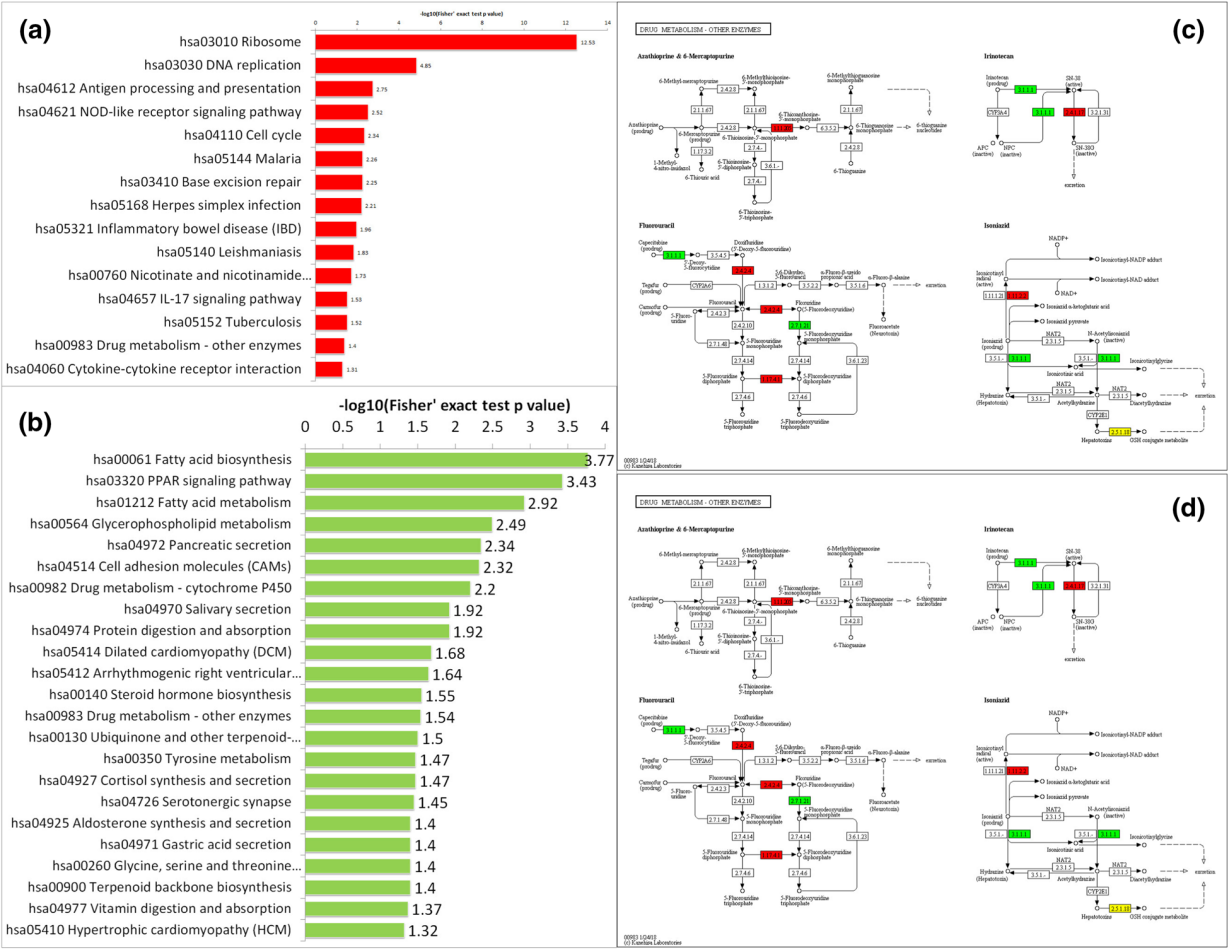
KEGG enrichment pathways (pso/con). **a** Upregulated DEPs. **b** Downregulated DEPs. The values on the horizontal axis are negative logarithmic conversions of significant P values (P < 0.05). **c** Drug metabolism-other enzyme pathways. **d** Drug metabolism-cytochrome p450 pathways

KEGG analysis was also applied with the Database for Annotation, Visualization, and Integrated Discovery (https://david.ncifcrf.gov/) to functionally enrich gene functions and to identify functional and metabolic pathways. As shown in Fig. 3, the DEPs were mainly enriched in 38 pathways. The upregulated DEPs were mainly enriched in the ribosomal pathway, DNA replication pathway, antigen processing and presentation, and the nucleotide oligomerization domain (NOD)-like receptor pathway, followed by the cell cycle pathway, base repair pathway, interleukin (IL)-17 pathway, drug metabolism pathway, cytokine receptor interaction pathway, malaria, inflammatory bowel disease, and herpes simplex infection (Fig. 3a). The downregulated DEPs were mainly assigned to the fatty acid synthesis pathway, fatty acid metabolism pathway, peroxisome proliferator-activated receptor (PPAR) pathway, glycerophospholipid metabolism pathway, cell adhesion molecule and drug metabolism-P450 pathway, followed by drug metabolism-other enzyme pathway, protein digestion and absorption pathway, cortisol synthesis pathway, pancreatic juice secretion pathway, saliva secretion pathway, and gastric juice secretion pathway (Fig. 3b).

### Cluster analysis of DEPs

The DEPs were divided into four groups for cluster analysis of GO and KEGG using the following |fold-change| as the cutoff thresholds: Q1 (0 < Ratio ≤ 1/1.5), Q2 (1/1.5 < Ratio ≤ 1/1.3), Q3 (1.3 < Ratio ≤ 1.5), and Q4 (Ratio > 1.5).

**Fig. 4:**
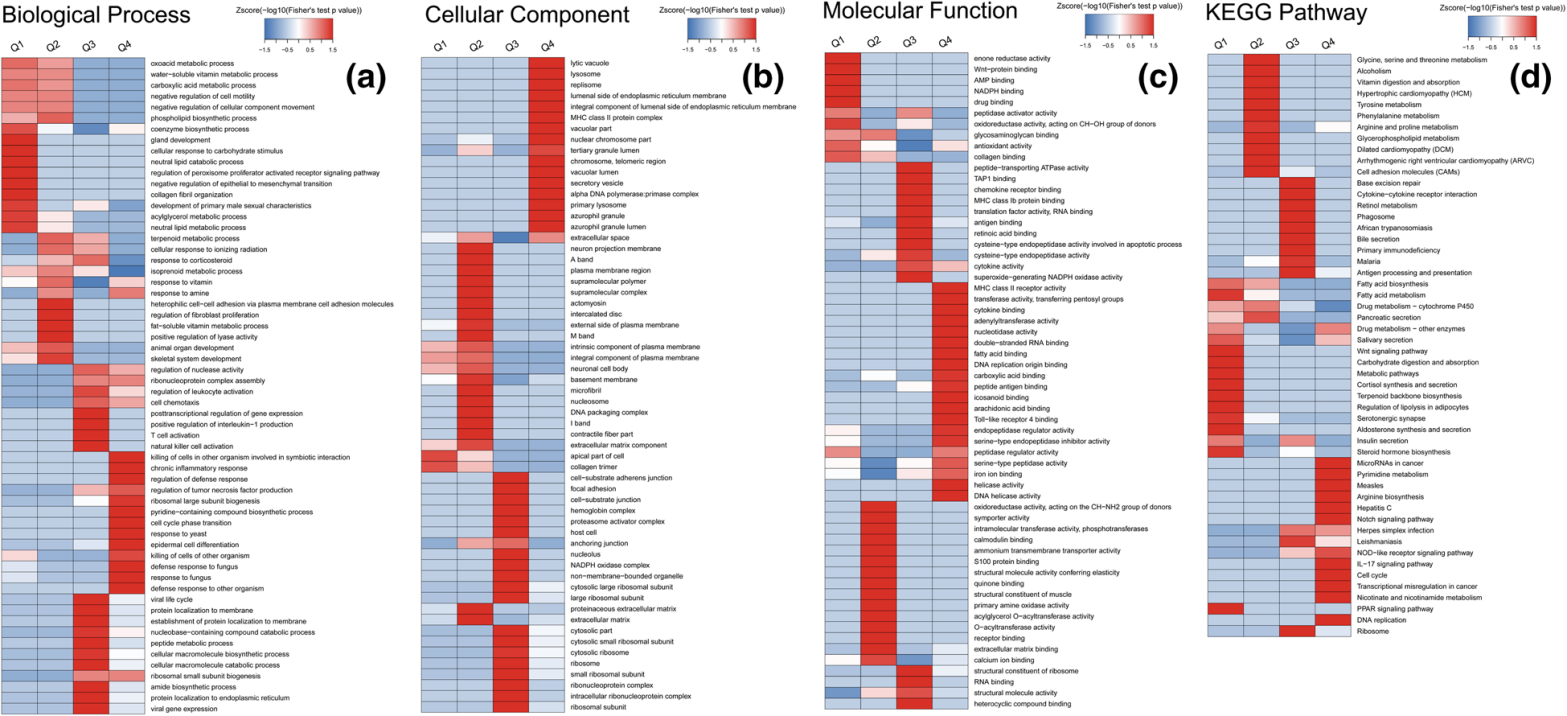
Heat map of cluster analysis for GO annotation and KEGG enrichment pathways. **a** GO term BP. **b** GO term CC. **c** GO term MF. **d** KEGG pathways

In the BP category, the GO terms of the upregulated DEPs were mainly enriched in protein synthesis, cell differentiation, and activation of inflammatory signaling pathways, such as chronic inflammatory response, T cell activation, NK cell activation, tumor necrosis factor (TNF) production regulation, synthesis of large nucleic acid subunits, cell cycle phase transition, epidermal differentiation, defense response, membrane localization of proteins, and synthesis and decomposition of cellular macromolecules. The downregulated DEPs were mainly related to cell proliferation, including cell responses to carbohydrates and the PPAR signaling pathways, collagen proliferation regulation, lipid metabolism process, cell adhesion molecule regulation, and cytoskeleton development (Fig. 4a).

As shown in Fig. 4b, in the CC category, the upregulated DEPs were mainly related to the body’s cellular immunity such as lysosomes, endoplasmic reticulum membrane space, MHC-II protein complex, nuclear chromosomes, telomere regions, DNA polymerase, and aniline blue particles. The downregulated DEPs were mainly enriched in the extracellular matrix, basement membrane, microfibers, supramolecular polymers, and nucleosomes, which may be closely related to keratinization and proliferation as well as dermal papillary capillary proliferation in the pathogenesis of PV.

In the CC category, the upregulated DEPs were mainly associated with immune defense, including MHC-type II receptor activation, antigen binding, cytokine activation and binding, transferase activity, nucleic acid activation, Toll-like receptor (TLR)-4 binding, helicase activation, and carboxylic acid binding. The downregulated DEPs were mainly related to PV epidermal proliferation such as collagen binding, extracellular matrix binding, and oxidoreductase activity (Fig. 4c).

KEGG cluster analysis revealed that the upregulated pathways included the ribosome pathway, DNA replication pathway, NOD-like receptor pathway, IL-17 pathway, cell circulation pathway, and drug metabolism pathway, which might be associated with protein synthesis, inflammatory activation, and cell proliferation. The downregulation pathways were mainly related to apoptosis, protein recognition, and energy metabolism, including fatty acid biosynthesis pathways, fatty acid biometabolism pathways, PPAR pathways, and glycerophospholipid metabolism pathways (Fig. 4d).

### PRM verification

**Fig. 5:**
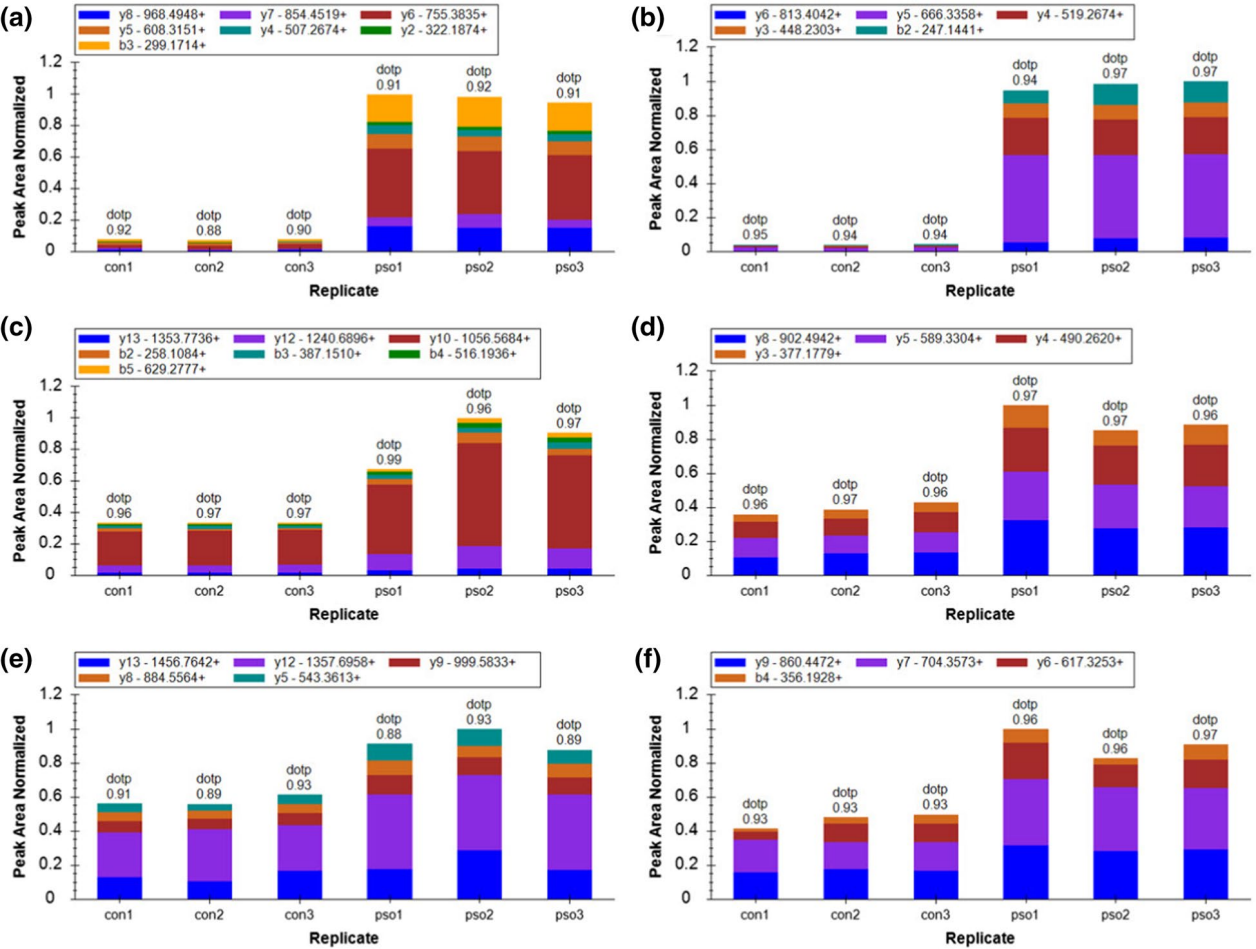
The PRM peak area of the selected DEPs. **a** Ion peak area distribution of the IANVFTNAFR fragment corresponding to MPO protein. **b** Ion peak area distribution of the VFFASWR fragment corresponding to MPO protein. **c** Ion peak area distribution of the EQEELLAPADGTVELVR fragment corresponding to TYMP protein. **d** Ion peak area distribution of the ALQEALVLSDR fragment corresponding to TYMP protein. **e** Ion peak area distribution of the LPIVNEDDELVAIIAR fragment corresponding to IMPDH2 protein. F. Ion peak area distribution of the VAQGVSGAVQDK fragment corresponding to IMPDH2 protein

Thirteen target proteins that are involved in two drug metabolism pathways (drug metabolism-other enzymes, drug metabolism-cytochrome P450 pathway) were validated by PRM, of which 10 were quantitated, and 9 of 10 were significant (Table 1). Among them, myeloperoxidase (MPO), thymidine phosphorylase (TYMP), inosine monophosphate dehydrogenase 2 (IMPDH2), glutathione S-transferase mu 4 (GSTM4), and aldehyde dehydrogenase, dimeric NADP-preferring (ALDH3A1) were highly expressed in PV; whereas carboxylesterase 1 (CES1), monoamine oxidase B (MAOB), microsomal glutathione S-transferase 1 (MGST1), and glutathione S-transferase theta-1 (GSTT1) were less expressed in PV tissues (Fig. 5). The three proteins MPO (p/c: 17.05, P < 0.05), TYMP (p/c: 2.44, P < 0.05), and IMPDH2 (p/c: 1.78, P < 0.05) in the drug metabolism-other enzyme pathways were significantly highly expressed in the PV lesional tissues (Fig. 5). The PRM results were consistent with those of TMT-LC–MS/MS, showing a significant increase in the fold change and further confirming the involvement of drug metabolism-other enzyme pathways in the pathogenesis of PV.

**Table 1:** PRM quantitative results of 9 DEPs

Protein accession	Protein gene	Con relative abundance	Pso relative abundance	Pso/con ratio	Pso/con ratio (TMT)	Pso/con P-value
P05164	MPO	0.11	1.89	17.05	2.04	2.92E−09
P19971	TYMP	0.58	1.42	2.44	1.66	7.02E−05
Q03013	GSTM4	0.66	1.34	2.04	1.63	1.04E−05
P12268	IMPDH2	0.72	1.28	1.78	1.35	1.25E−04
P30838	ALDH3A1	0.80	1.20	1.50	1.41	1.56E−05
P27338	MAOB	1.24	0.76	0.62	0.71	9.81E−03
P10620	MGST1	1.29	0.71	0.55	0.54	1.63E−03
P23141	CES1	1.42	0.58	0.41	0.59	4.86E−05
P30711	GSTT1	1.57	0.43	0.28	0.41	3.62E−06

## Discussion

Psoriasis is a chronic autoimmune inflammatory disease associated with multiple metabolism disorders of the epithelium that is regulated by the interactions between keratinocytes, dendritic cells, and T lymphocytes. The skin is the body’s first line of defense and participates in innate immune responses by activating surface receptors including TLRs and NOD‑like receptors [5]. Previous studies have demonstrated strong associations between PV and various biological processes such as cell proliferation, apoptotic regulation, cell defense response, and oxidative stress. The cellular innate and adaptive immune responses, especially the activation of T cells, play a critical role in the pathogenesis of PV. Although there is no cure for psoriasis yet, many treatments can greatly reduce symptoms. Cyclosporine A, an immunosuppressive agent that inhibits T cell proliferation and cytokine production, was the first clinical agent used to treat psoriasis, and the clinical data suggest a potential role of T cells in the pathogenesis of psoriasis. Recently, T cell-targeted monoclonal antibodies such as the anti-IL-17A agent secukinumab, anti-CD4 monoclonal antibody, and cytotoxic T lymphocyte-associated antigen 4-immunoglobulin have demonstrated significant therapeutic efficacy in the treatment of psoriasis [13]. Additionally, proteomic-based platforms have been emerging as increasingly powerful methods to identify both the potential mechanisms and biomarkers of disease pathogenesis. We identified 4404 DEPs that were mainly involved in the oxidative phosphorylation pathway, the tricarboxylic acid cycle, and the NF-κB and RAS signaling pathways in our previous study. Western blot confirmed higher expression levels of P50/P65/IKKβ in the lymphocytes of PV patients compared with those of heathy controls [11]. The proteins identified by LC–MS/MS, including SFN, GSTP1, FABP5, S100A7, and RABP2, were also observed to be upregulated in psoriasis lesional tissues (Additional file 6: Table S3) [14, 15].

To the best of our knowledge, the current study revealed a strong association between protein expression in psoriasis lesions and drug metabolism pathways for the first time. The proteins identified in our previous study, including MPO, TYMP, IMPDH, ALDHs, and GSTT1, were also highly expressed in this study, which indicated that drug metabolism played an important role in the pathogenesis of psoriasis. PRM validation of 9 proteins that were highly expressed in the drug metabolism pathway further confirmed significant upregulation of MPO (17.05), TYMP (2.44), and IMPDH2 (1.78) in psoriasis lesions. These proteins are closely related to the activation of T cells and neutrophils, among which MPO is an indicator of neutrophil function and activation [16]. It has been shown that neutrophil phagocytic activity is reduced in MPO-deficient patients with generalized pustular psoriasis [17], leading to a small degree of infection compared with the MPO-normal patients. MPO has been proven to play a key role in aggravating the inflammatory response and tissue damage in PV patients. In addition, TYMP has been demonstrated to directly stimulate the expression of angiogenic factors such as VEGF, TNFα, and IL-8 [18] as well as promote angiogenesis [19, 20]. Moreover, IMPDH2, which is synthesized by guanine nucleotide rate-limiting enzymes, provides guanine nucleotides for T cell proliferation [21, 22] and is highly expressed in proliferating tumor tissues [23]. The function and roles of TYMP and IMPDH2 and other proteins participating in the drug metabolism pathway in the pathogenesis of PV are unknown and need to be further studied.

The present study identified 505 DEPs, among which upregulated DEPs were mainly enriched in the processes of nucleic acid binding, RNA binding, ribosome activation, and endoplasmic reticulum protein synthesis, suggesting that PV involves the proliferation of keratinocytes [24]. The downregulated DEPs were mainly assigned to the processes of extracellular matrix structural composition, protein complex binding, collagen binding, and cell lipid metabolism, indicating a reduction in the synthesis of psoriatic extracellular matrix. The extracellular matrix consists of the basal cells anchoring the basement membrane. The reduction in the synthesis of extracellular matrix might be related to the epidermal remodeling of psoriatic lesions [25]. Furthermore, the pathogenesis of PV is related to the activation of a variety of immune cells. In the present study, DEPs were significantly enriched in a variety of immune cell activation processes, including T cell activation and leukocyte activation, and immune cell composition processes such as aniline blue granule synthesis, antibacterial peptide synthesis, S100 protein binding, TNF production regulation, and cytokine activation and binding. KEGG pathway enrichment analysis revealed significant associations between several pathways and the pathogenesis of PV, including upregulation of the NOD-like receptor pathway, antigen processing and presentation, inflammatory bowel disease, the IL-17 pathway, and cytokine receptor interactions as well as downregulation of the PPAR pathway, fatty acid metabolism, cell adhesion molecules, and protein digestion and absorption. Recent studies also have confirmed the associations between these pathways and occurrence and development of PV [26–29].

## Conclusion

In conclusion, the present study successfully identified a set of DEPs with quantitative proteomic technologies. Nine proteins (MPO, TYMP, IMPDH2, GSTM4, ALDH3A1, CES1, MAOB, MGST1, and GSTT1) are potential biomarkers of PV pathogenesis. These findings provide novel insight into the pathogenesis of PV for the future investigation of immunotherapy techniques.

## Supplementary information

**Additional file 1: Table S1.** Samples information.

**Additional file 2: Fig S1.** Details of patients’ pathological sections.

**Additional file 3: Fig S2.** Mass error and peptide length.

**Additional file 4: Fig S3.** PRM mass spectrogram.

**Additional file 5: Table S2.** Quantitative information and annotation terms of 3648 proteins.

**Additional file 6: Table S3.** Venn diagram and same proteins.

## Data Availability

All data generated or analyzed during this study are included in this published article and its additional files.
