# Supplementary figures and images for: Quantitative analysis of differentially expressed proteins in psoriasis vulgaris using tandem mass tags and parallel reaction monitoring
Source: Clin Proteomics. 2020 Aug 12;17:30. doi: 10.1186/s12014-020-09293-8 (PMC7425065; doi:10.1186/s12014-020-09293-8)

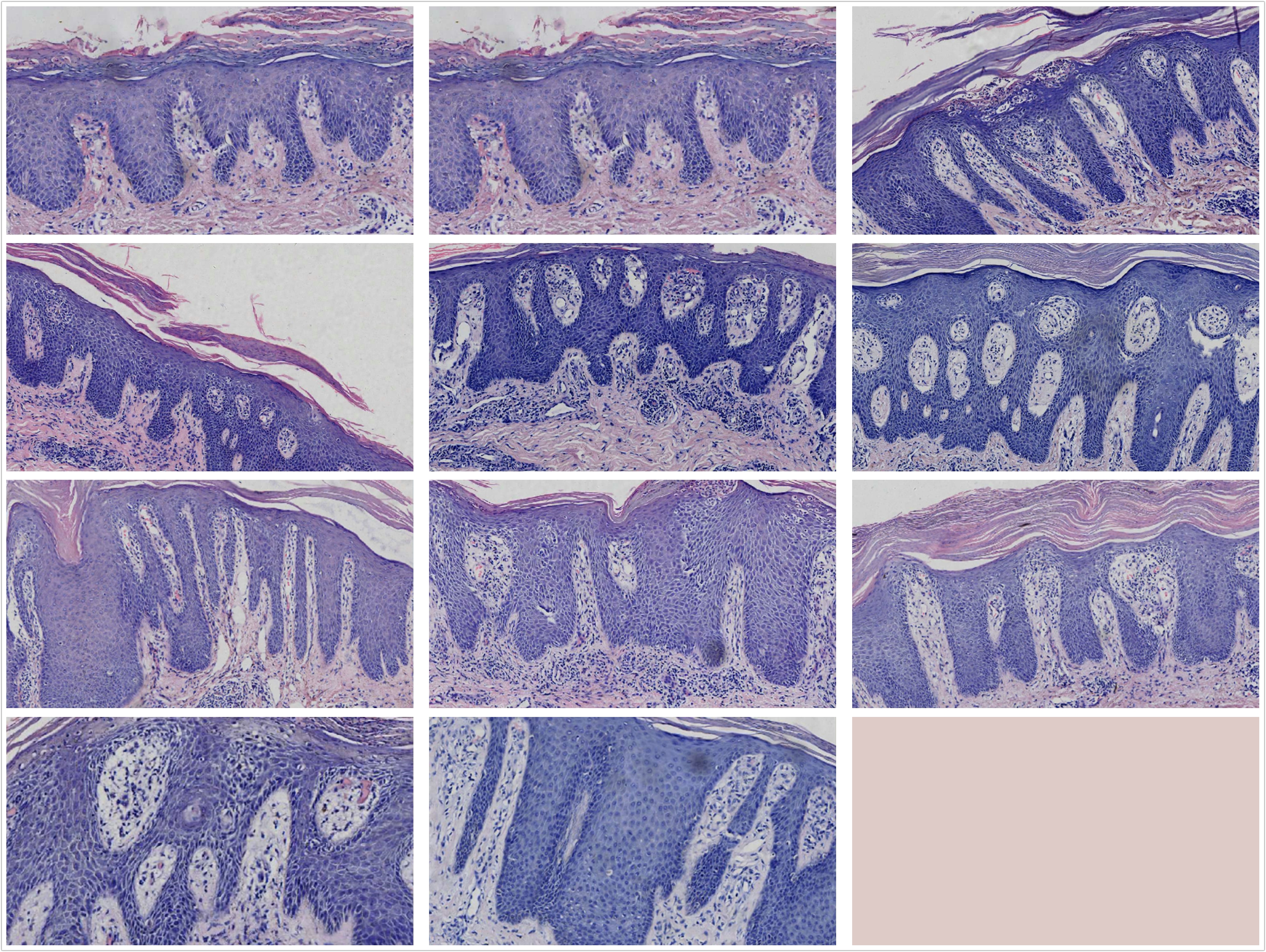

Supplement: Supplementary file 2 — Additional file 2: Fig S1. Details of patients’ pathological sections. [file 12014_2020_9293_MOESM2_ESM.jpg]

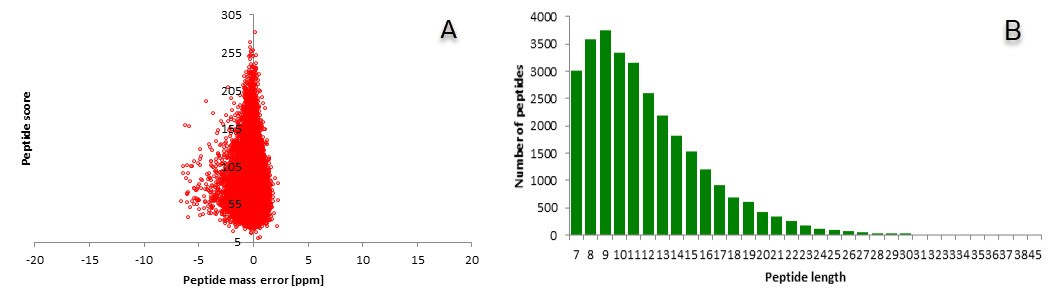

Supplement: Supplementary file 3 — Additional file 3: Fig S2. Mass error and peptide length. [file 12014_2020_9293_MOESM3_ESM.jpg]

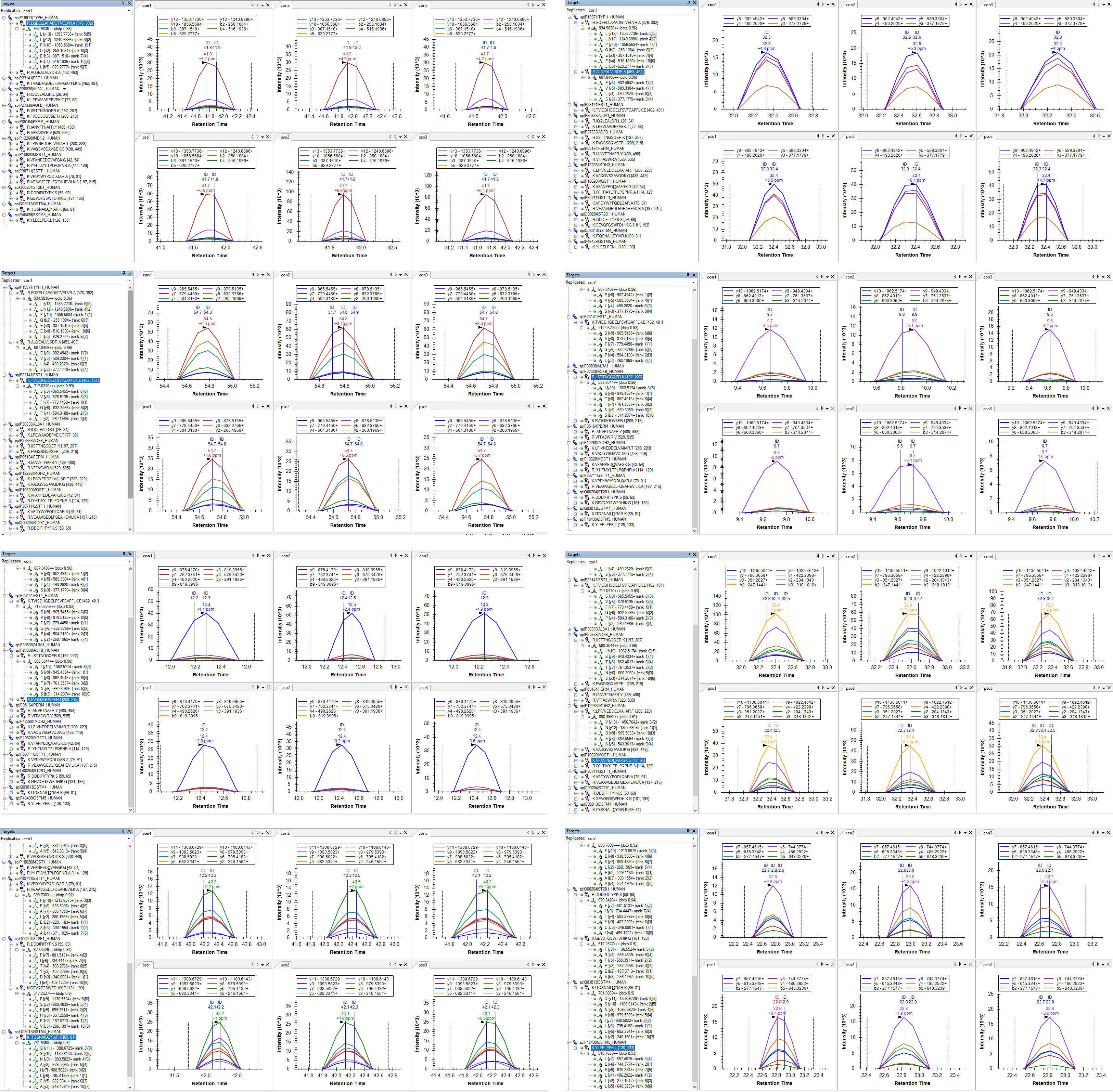

Supplement: Supplementary file 4 — Additional file 4: Fig S3. PRM mass spectrogram. [file 12014_2020_9293_MOESM4_ESM.png]
